# Developing the Resident Measure of Safety in Care Homes (RMOS): A Delphi and Think Aloud Study

**DOI:** 10.1111/hex.13730

**Published:** 2023-02-16

**Authors:** Natasha Tyler, Claire Planner, Bethany Shears, Andrea Hernan, Maria Panagioti, Sally Giles

**Affiliations:** ^1^ National Institute for Health and Care Research Greater Manchester Patient Safety Translational Research Centre, Division of Population Health, Health Services Research and Primary Care, Manchester Academic Health Science Centre University of Manchester Manchester UK; ^2^ National Institute for Health and Care Research School for Primary Care Research University of Manchester Manchester UK; ^3^ Leeds Teaching Hospitals NHS Trust Leeds UK; ^4^ Centre for Primary Care and Health Services Research University of Manchester Manchester UK; ^5^ Faculty of Health Deakin University Warrnambool Australia

**Keywords:** care homes, consensus methods, contributory factors to safety, Delphi, nursing homes, older adults, patient‐reported safety

## Abstract

**Objective:**

This study aimed to develop a measure of contributory factors to safety incidents in care homes to be completed by residents and/or their unpaid carers.

**Introduction:**

Care home residents are particularly vulnerable to patient safety incidents, due to higher likelihood of frailty, multimorbidity and cognitive decline. However, despite residents and their carers wanting to be involved in safety initiatives, there are few mechanisms for them to contribute and make meaningful safety improvements to practice.

**Methods:**

We developed 73 evidence‐based items from synthesis and existing measures, which we presented to a panel of stakeholders (residents/carers, health/social care professionals and researchers). We used two online rounds of Delphi to generate consensus (80%) on items important to include in the Resident Measure of Safety in Care Homes (RMOS); a consensus meeting was later held. The draft RMOS developed through the Delphi was presented to participants during ‘Think Aloud' interviews using cognitive testing techniques.

**Results:**

The 29‐item RMOS was developed. Forty‐three participants completed Delphi round 1, and 27 participants completed round 2, 11 participants attended the consensus meeting and 12 ‘Think Aloud’ interviews were conducted. Of the 73 original items, 42 items that did not meet consensus in Delphi round 1 were presented in round 2. After the consensus meeting, it was agreed that 35 items would comprise the RMOS questionnaire and were presented in the ‘Think Aloud’ interviews. Participants suggested numerous changes to items mostly to improve comprehension and ability to answer.

**Conclusion:**

We have a developed an evidence‐based RMOS, with good face validity, to assess contributory factors to safety in care homes from a resident/carer perspective. Future work will involve psychometrically testing the items in a pilot and developing a complementary simplified, dementia‐friendly version to promote inclusivity.

**Patient or Public Involvement:**

Four patient and public contributors worked with researchers to develop the online questionnaires. Patients (residents) and carers participated on the consensus panel. One member of the research team is an expert by lived experience and was involved in design and analysis decisions. The item list and instructions for the questionnaires were reviewed for face validity, understanding and acceptability by a patient and public involvement group and modified.

## 1 INTRODUCTION

Ensuring patient safety in care homes is a longstanding challenge in the United Kingdom, which has been further evidenced to the public during the SARS‐CoV‐2 pandemic.^1^ This is complicated by the fact that older residents in care homes often live with long‐term conditions and functional decline, take multiple medications and experience recurrent hospitalizations.^2^ Care home residents are particularly vulnerable to experiencing patient safety incidents, which can lead to severe but avoidable harms including hospital re‐admissions, infections, medication harm and mortality.^3^ They are twice more likely to experience emergency admissions than the rest of the population over the age of 75 years, and 40% experience adverse events within 45 days after their discharge from hospitals back to care homes.^4^

A recent publication from WHO suggests that improving patient safety should begin with engaging patients.^5^ In primary and secondary care settings, evidence suggests that patients are able to identify the factors that contribute to patient safety incidents. These contributory factors are often defined as proximal and latent causes of error.^6^ Examples include ineffective communication between professionals, poor quality of treatment or inappropriate physical environments.^6^ Capturing patient feedback about contributory factors to patient safety incidents and using this feedback for making safety improvements is a novel and promising field of research. It is known that patients, carers and family members are able to provide a different and valuable perspective on patient safety in hospitals and primary care; however, less is known about the care home setting.^7^, ^8^, ^9^, ^10^ A measure of patient feedback on safety is therefore needed in this setting. This would ensure that resident and carer preferences are accounted for, and provides another measure for safety outside routinely collected data and case record reviews.^11^ Patient safety improvement projects in the UK care homes sector could considerably benefit from engaging residents and their relatives.^12^ Enabling residents to provide feedback on safety has the potential to improve person‐centred care, which is now embedded in care home practice and is a central component of quality and safety in health services.^13^

Feedback measures for assessment of factors contributing to safety have successfully evaluated safety improvement interventions utilizing patient feedback in other clinical settings.^7^, ^8^, ^9^, ^10^ These measures have proved acceptable and effective in improving patient safety in primary care and hospital settings in the United Kingdom and internationally. For example, the Primary Care Patient Measure of Safety (PC PMOS) was designed to facilitate learning among staff members and organizations based on patient feedback, and drive implementation of real‐time service improvements, embracing the view that healthcare staff are more likely to engage with interventions that they have been involved in developing.^14^

At present, no measures utilize residents' perspectives of contributory factors when making safety improvements for older people/their carers living in residential care homes.^15^ Although a proportion of older residents in care homes might not be able to engage with a self‐reported patient feedback measure due to advanced cognitive decline, most residents and/or their carers will be able to provide some level of feedback on patient safety and can play a vital role in the success and longevity of patient safety improvement interventions in care homes. A theory‐driven, evidence‐based measure, derived from James Reason's Swiss Cheese model of accident causation theory, which identifies the latent conditions (contributory factors) responsible for patient safety incidents,^16^ and similar to the PC PMOS would be particularly helpful to capture information about the contributory factors to patient safety in care homes from the residents'/carers' perspective. The aim of this study was to develop a resident/carer measure of safety that can be used as a basis for proactively preventing and improving safety in care homes.

## 2 METHODS

### 2.1 Study overview

**Figure 1:**
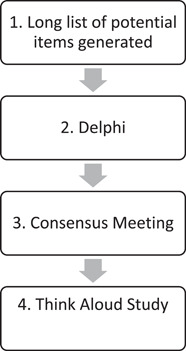
Summary of methods.

The Resident Measure of Safety in Care Homes (RMOS) was developed in four stages, and involved residents, carers and healthcare professionals at each stage: (1) a long list of potential items was generated from conducting a systematic literature review^17^ and through exploration of existing measures; (2) the list was presented to stakeholders in an online Delphi process (2 rounds); (3) the results of the Delphi survey were appraised at a consensus meeting; and (4) the resulting measure was presented to a small group of participants in a ‘Think Aloud Study’ for face validity testing; see Figure 1.

### 2.2 Participants

Participants (from the United Kingdom) were recruited in several different ways. Academic researchers were recruited if their research had been included in our systematic review;^17^ in addition, known researchers in the field identified by the team were also approached. They were approached by email. Care Home residents were recruited through a ‘gatekeeper’ in three care homes. ‘Gatekeepers’' were care home managers who identified and approached residents. Information leaflets were shared and residents self‐identified. We asked participants to read the information sheet and to summarize it; this was used to assess capacity, and those who could not summarize it were excluded. We recruited a number of care home professionals through these sites. Sites were selected to represent different demographic areas; however, most care homes did not have the capacity to take on any research during covid, so this limited our choice. The remaining healthcare professionals, care home professionals and carers were recruited through our teams' existing professional networks and social media (Twitter and Facebook). One author (B. S.) also emailed multiple UK care homes registered on www.carehome.co.uk. Participants were eligible to participate if they had personal or professional experience of transfers between care homes and hospitals.

The same participants were involved throughout the Delphi research process. Consensus meeting participants who had completed both Delphi rounds were asked to indicate whether they would be interested in attending a face‐to‐face meeting. We invited a random sample of interested participants to attend, while ensuring representation of all key stakeholder groups. If a participant declined the invitation to the consensus meeting, a similarly matched participant was invited from the Delphi panel. They were selected based on their stakeholder view, i.e., care home staff, family member or patient safety expert.

### 2.3 Stage 1: Gathering information

To develop our initial pool of items, we synthesized items derived from existing evidence in this field and items from exiting measures in other settings. Our systematic review of contributory factors to safety in care homes,^17^ which informed this work (submitted), identified 18 contributory factors with two additional categories that were added to the existing contributory factors to the safety in hospitals framework^9^ (family‐related factors and organization and care planning). Items from two existing patient‐reported outcomes measures (developed for hospitals and primary care)^8^, ^18^ were combined to generate a long list of potential items. This list was discussed by three authors N. T., S. G., B. S.) in a structured meeting. Each potential item was considered in turn and each member had the opportunity to present arguments for or against inclusion. For each item, it was decided whether the item should be a stand‐alone item, edited for the care home setting (e.g., we adapted from the PMOS, ‘the hospital departments were very clean’ to ‘the care home was very clean’), combined with another item of a similar thematic nature or removed if not relevant to a care home setting (e.g., ‘I was able to make an appointment at a time that suited me’). If all three authors agreed to remove or merge an item, it was amended; otherwise, the item remained as a stand‐alone item.

### 2.4 Stage 2: Delphi survey

The Delphi technique is a research method aimed at generating consensus.^19^, ^20^ It solicits opinions from stakeholder groups in an iterative process of answering questions about which items should be included and why. After each round, the responses are summarized and redistributed for discussion in the next round. We chose a priori to hold two Delphi rounds in this study. The item list that was finalized after group discussion in stage 1 was used to populate the first Delphi questionnaire. Any items without consensus after the first round were re‐presented in round 2.

Surveys were distributed to participants who consented to take part. For contacts in the researchers' networks and those recruited using social media, this was via an online link. For residents, these were in paper format due to lack of technology in care homes. Managers also distributed electronic copies via email to staff. Round 1 remained open for 1 month (9 February to 10 March 2021) and participants received two follow‐up reminder emails. Round 2 was open between 15 March and 12 September due to issues with contacting care home residents during the SARS‐CoV‐2 pandemic (see Section 4.1). We conducted the Delphi survey using Qualtrics, a secure online hosting platform.^21^ After reading a participant information sheet and giving informed consent (by ticking a box), participants selected their stakeholder group. Participants answered a number of demographic questions, gender, ethnicity, etc, and self‐generated an identification code for follow‐up.

In each round, participants were asked whether the items should become part of the RMOS measure. A 9‐point Likert scale was used: ranging from (9) Highly relevant to (1) Highly irrelevant; items 8–2 were presented as numerical values with no text labels. There was a free‐text comments box and participants were encouraged to provide comments that would be fed back anonymously to the whole group. Participants could suggest additional items/contributory factors to safety at the end of round 1, which were reviewed by the core research team. Any contributory factor not already represented was added to round 2.

In round 2, median group scores for each item and anonymous comments for and against from the previous round were presented, and participants were asked to reflect on the information and score each item again. The percentage of participant agreement with each item on a scale of 1–9 was calculated from the scores obtained during round 1 and again in round 2.

The literature suggests that consensus levels should be set a priori at a minimum of 70%.^22^, ^23^ We unanimously chose an 80% consensus level to increase sensitivity. Consensus criteria were defined a priori: items scored as relevant or highly relevant (7–9) by 80% or more of the group reached consensus for inclusion and were included in the first RMOS draft. Items scored as irrelevant or highly irrelevant (1–3) by 80% or more were defined as having reached consensus for exclusion and were excluded. Items with >80% agreement but many comments were sent back to the panel in round 2 to improve readability. Items not fulfilling criteria for consensus inclusion or exclusion were defined as not having reached consensus and were therefore re‐presented in round 2 for reconsideration. Decisions for inclusions and exclusion criteria were made a priori to reduce burden between rounds by removing items that fulfil criteria. We decided a priori to limit the process to two Delphi rounds due to the complexities of conducting research during the Covid‐19 pandemic; stopping a Delphi after a predetermined number of stages is common in the existing literature.

### 2.5 Stage 3: Consensus meeting

The results of the Delphi survey were presented at a consensus meeting. The main goal of the consensus meeting was to decide which items will be included in the first draft RMOS. This was chaired by four members of the team (N. T., S. G., C. P., M. P.) with expertise in consensus methodology and patient involvement and engagement. Participants were sampled to achieve a balanced representation of carers, healthcare professionals, care home professionals and researchers. Unfortunately, due primarily to Sars‐CoV‐19 restrictions on care home access, we could not recruit residents to this component. We aimed to have a small representative group of between 9 and 12 participants to enable meaningful small group discussions, similar to previous consensus meetings.^24^, ^25^

The format of the consensus meeting comprised of (a) a short overview of the study and (b) a summary of the Delphi results sorted by stakeholder group, beginning with the items that were agreed to be included after round 2 of the Delphi, any items that might be contentious and finally all of the remaining items presented in round 1.^26^ Participants were asked if there were any fundamental reasons why these items should not be included in the RMOS measure. Divergent views were actively sought and the chair ensured that everyone had the opportunity to participate in discussions. Data were collected to ascertain which items should proceed to the think aloud stage and reasons for exclusion or reintroduction.

### 2.6 Stage 4: Think Aloud

A selected sample of participants was then asked to complete ‘Think Aloud’ interviews. This involved completing the RMOS and describing their thought process when arriving at their answer. Three researchers conducted the ‘Think Aloud’ interviews, using a standardized prompt sheet that provides example ‘Think Aloud’ questions to familiarize participants with the process and then questions about the items covering 1. Comprehension, 2. Retrieval, 3. Judgement and estimation, 4. Documenting a response and standardized associated prompts (i.e., can the participant make a judgement based on the information that they are able to retrieve). Think aloud methodology was based on work by Tourangeau.^27^ Interviews were audio‐recorded.

From an analysis perspective, each interviewer coded each item (for each participant) for 1. Comprehension and 2. Ability to answer (based on retrieval/judgement/estimation/documenting a response) into one of three categories (Yes, no, partially). An overall percentage score based on comprehension and ability to answer the question was calculated for each item and then each item was discussed in person at a team meeting (S. G., C. P., N. T.) to generate the final version of the RMOS presented in this paper.

### 2.7 Patient and public involvement (PPI)

Four patient and public contributors worked with researchers to develop the online questionnaires. Patients (residents) and carers were also represented alongside professionals and researchers in the consensus panel. One member of the research team is an expert by lived experience and was involved in design and analysis decisions. The item list for round one and instructions for the questionnaires were reviewed for face validity, understanding and acceptability by a PPI group and modified according to feedback. All public contributors were remunerated for their time.

## 3 RESULTS

### 3.1 Stage 1: Item generation

Our systematic review has been described in detail elsewhere.^17^ In summary, 24 items were identified from 67 studies. The studies identified a wide range of factors contributing to the safety of care home residents. These factors were hierarchically ordered from proximal (sharp end), or those closest to the patient safety incident (e.g., individual task performance) to distal (latent), or those furthest away from the patient safety incident in care homes (e.g., ineffective systems and processes). The five most common safety domains from the review were task performance, team factors, staff training and education, communication and organization and care planning; see Supporting Information: File 3. In addition, 49 items were taken from the PC PMOS and PMOS, which were evidence‐based validated measures developed with patients and experts for other care settings, 27 of which were adapted for a care home setting by the research team. After discussion within the research team, 73 items were taken forward into the Delphi process.

### 3.2 Stage 2: Delphi process

Forty‐four participants completed round one of the Delphi (19 residents and carers, 9 researchers/policy makers and 16 healthcare and care home professionals) and 27 participants completed round two (9 residents and carers, 8 researchers and 10 healthcare and care home professionals). Seventeen participants dropped out after round 1; 11 were residents. There was a 39% attrition rate between rounds 1 and 2 of the Delphi, which we attributed to the effects of the SARS‐CoV‐2 pandemic and the participant group.

After round 1, 42 items fulfilled the criteria for inclusion in round 2; 31 were excluded. Of the 42 items presented again to the group in round 2, 8 fulfilled the criteria for exclusion. Therefore, a list of 34 items that were agreed upon was presented to the group in the consensus meeting. Supporting Information: File 1 shows consensus levels for each outcome in each round.

### 3.3 Stage 3: Consensus meeting

Twelve participants attended the consensus meeting, with equal representation from each of the three categories: four participants were researchers, four identified as carers/relatives and four identified as healthcare/care home professionals.

Each of the items was considered individually and discussions indicated that many could be made clearer. Subsequently, twenty‐one items remained the same, 2 items were combined, 12 items were adapted and 3 items were excluded. Four items that were excluded in round 1 and 2 items that were excluded in round 2 were discussed in the consensus meeting and reintroduced either through combination with an existing item or by adapting the wording. Thirty‐five items were presented in the ‘Think Aloud’ study.

### 3.4 Stage 4: Think Aloud

Twelve participants completed the ‘Think Aloud’ interview. Nineteen items were adapted as a result, which typically involved changing one or two words, for example, ‘my friend/relative is aware of how to report a concern' was changed to ‘we are aware of how to report a concern’. Nine items remained the same and six items were removed. Most items were removed because participants felt that they were not able to make an adequate judgement to sufficiently answer the question, for example, ‘other health care professionals know enough about my relative/friends health conditions’. Our ‘Think Aloud' process indicated that the measure would be most useful for carers and residents with capacity. Supporting Information: File 2 shows the results and decisions made as a result of the Think Aloud process.

**Figure 2:**
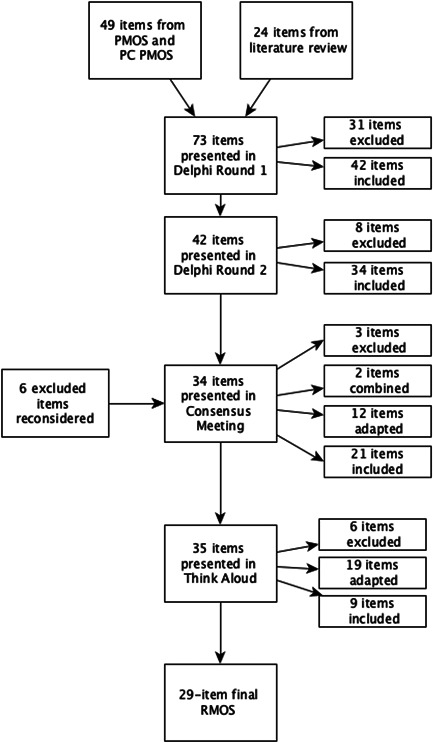
Results.

The final PMOS measure developed after the Think Aloud interviews is presented in Figure 2, Table 1.

**Table 1:** Final RMOS tool and included items.

Item number	Final Item
1	Residents are supported to contact their family or friends
2	Residents are involved in care
3	Residents are always treated with dignity and respect
4	There are enough staff to look after the needs of residents
5	Things get done in a timely manner (e.g., help with going to the toilet)
6	There is always a member of staff available with the knowledge/skills to perform specific tasks
7	Residents are always given the right food for them
8	The care home is very clean
9	The physical environment of the care home feels safe (e.g., good lighting levels, enough space, no clutter)
10	All equipment needed for residents' care always work properly
*These statements are about the staff that work in the care home (e.g., carers, nurses, nursing assistants, managers) rather than staff that visit from outside agencies*	
*Care Home Staff…*	
12	…listen to residents
13	…always know important information about residents' care
14	…interact with my residents in an acceptable manner
15	.communicate well with one another
16	… use language that is offensive or makes residents upset or frightened
17	… wear appropriate PPE (personal protective equipment) when interacting with residents (e.g., gloves, apron, face masks)
18	… always have access to information about residents' needs
19	… are always able to get advice from doctors and nurses when needed
*These statements are about sharing information*	
20	We are informed of mistakes made
21	We have been informed of any infection outbreaks and the action to control further spread
22	We are always offered enough information about their care
23	We always receives answers to all the questions we have regarding care
24	We are encouraged and supported to be involved in decisions about care
25	We are aware of how to raise a concern about a member of staff
26	Healthcare professionals who visit the care home have all the necessary information
*These statements are about hospital visits*	
27	Residents are able to attend hospital appointments when required
28	Care home staff had information about the most recent hospital visit (e.g., test results or medication changes)
29	The GP had information about the most recent hospital visit (e.g., test results or medication changes)

## 4 DISCUSSION

This paper describes the development of the RMOS. This measure facilitates the involvement of residents and carers in managing safety and has the potential to improve the quality and safety of care provided within residential care homes. The measure provides a vehicle to enable patient and carer voices to be heard and acted upon in a standardized way. Staff in care homes can use the feedback to develop targeted interventions based on the feedback that they receive. Similarly to the PC PMOS (its primary care counterpart),^18^ PMOS (the secondary care version), the RMOS encompasses a wide range of domains that are considered to be both error‐producing and latent contributory factors to safety. These include contributory factors identified in a recent systematic review^17^ such as performance, team factors, staff training and education, communication and organization and care planning. A strength of the measure is that it enables self‐report of factors that can contribute to safety incidents and therefore takes a more upstream and preventative view of safety in the care home environment rather than a reactionary approach that other post‐incident measures commonly use.

The RMOS is the first patient/resident‐reported measure of patient safety in care homes. A staff measure exists: the AHRQ Nursing Home Survey on Patient Safety Culture.^28^ Whilst some items in the RMOS show overlap with themes in this measure, such as communication openness and response to mistakes, further research is needed to assess how patient‐reported and professional‐reported measures of safety correlate in care home settings. Using the two measures in parallel would provide a more holistic picture of safety in care homes from multiple perspectives.

The online stakeholder panel agreed that 42 items should be included; after considerable discussion in the consensus meeting, 35 items remained and 29 items remained after the ‘Think Aloud’ process. The final version of the RMOS has established face validity and the items were agreed by stakeholders using consensus methods.

The Academic Health Science Network report ‘Improving Safety in Care Homes' called for three key changes for transforming safety in care homes,^29^ one of which was ‘to engage in proactive and systematic needs identification followed by effective interventions to optimise the care we provide’. The RMOS provides a vehicle to systematically identify safety needs from a resident/carer perspective at both the local and national level. The RMOS may be useful and practical for identifying areas of weakness and to plan continuous safety improvements, and complement current data collection methods to identify and prevent safety incidents in care homes. The RMOS can also be used as an intervention for care home staff to utilize real‐time resident and carer feedback and monitor change over time. The RMOS has a great potential to impact policy and practice by preventing safety incidents in care homes.

Figures suggest that approximately 70% of care home residents have dementia.^30^ Therefore, a large proportion of residents may lack the capacity to use this measure. Despite this, evidence suggests that residents with dementia and/or their carers would like to be involved in research and quality and safety initiatives. Stakeholder work suggests that improvements can be made to measure to enable inclusivity, such as limited response options and simplification/reduction of items.^15^, ^31^ Thus, the development of the RMOS is an important first step to identify what needs to be measured to make a meaningful patient‐reported safety dementia‐inclusive measure. It enables residents' voices to be heard, and is in line with multiple stakeholder and policy briefs,^15^, ^31^ which suggest that we need to work with residents and informal carers to identify how to support their involvement in improving the quality and safety of their care. The ‘Think Aloud’ study and consensus meeting indicated that making a complementary dementia‐friendly version of the measure is necessary. Novel examples, such as generating visual art (graphic vignettes) to represent questions and responses and conducting walking interviews to collect data, have been proven to be successful.^32^, ^33^

The key concern was regarding the ability of carers to make judgements about some contributory factors to safety that they have not witnessed or experienced. Examples of these domains/items include how staff communicate/work with one another or things that happen infrequently, such as abusive language. Many carers agreed that the SARS‐ CoV‐2 pandemic social‐contact limitation policy reduced their ability to make judgements about contributory factors to safety. It was also agreed that these items would be difficult for many carers to answer when social‐contact limitations are lifted, based on visits to a care home. Therefore, items that would be difficult for most carers to answer for this reason were removed following the ‘Think Aloud' process.

### 4.1 Limitations

The key limitation of this study was the attrition rate: 39% after round 1. Although attrition is very common in Delphi studies,^34^ we found the attrition rate for this study to be more pronounced than that in other Delphi studies.^35^, ^36^ We attribute the attrition rate to a number of factors, the first being the SARS‐CoV‐2 pandemic and associated governmental restrictions, which meant that access to care homes during the study period was not permitted; even the carers who contributed to the study reported only seeing their relatives for 30 min a week. Although several gatekeepers (care home volunteer/managers) circulated the paper versions of round 1 to care home residents, they were unable to collect/oversee the round 2 responses due to employment changes, policy changes and resource constraints. Also, recruiting residents who were able to participate proved difficult as many had dementia, sight or movement disorders. Therefore, we lost contact with the majority of the residents who participated in round 1 and a number of the professionals. Another limitation is the relatively small sample sizes and limited diversity within the sample.

The SARS‐CoV‐2 pandemic may not have affected other participants who responded online, and attrition rates for the online response group were very low. It is likely that using a paper version of a Delphi in the care home would result in future attrition implications. Therefore, finding solutions that reduce digital exclusion in an online‐only method would be beneficial and arguably easier to implement when there are no visiting restrictions. The implications of social distancing and the impact of the pandemic on care homes also meant that residents were not involved in all stages of the process; however, carers were involved in every stage.

In comparison with measures developed for other settings (primary and secondary care), during our stakeholder engagement and consensus process, concerns were raised about the appropriateness of items that measure safety in care homes in the measure's current format. The  stakeholders key concern was the ability of residents without capacity to use the measure (due to later stage dementia or organic mental health problems) to use the measure was considered problematic. Our ‘Think Aloud’ process and stakeholder consultation work indicated that the measure would be most useful for carers and residents with capacity and using cognitive testing in the ‘Think Aloud’ was a strength of this study. Also, stakeholders involved in the study found it difficult to make judgements on some key elements of safety, such as medication management, and as a result, this measure may not cover all elements of safety.

### 4.2 Future work

This study established consensus about the items that should be included in the RMOS measure. Face validity of the measure was also determined. Future research is needed to establish the psychometric properties of the RMOS measure and make targeted adaptations. This will result in an RMOS that can be used for residents with capacity and their informal carers to improve safety in care homes. In parallel, we recommend that the RMOS be refined into a complementary dementia‐friendly simplified version that can be used by people who might lack capacity, due to dementia or other cognitive decline. A dementia‐friendly version should be developed in combination with appropriate methods for data collection such as displaying the items as visual graphics, in line with recent advances in inclusive dementia research.^33^ The measure also has potential to be used by agencies that assess the quality and safety of care homes such the Care Quality Commission.

### 4.3 Conclusion

To the authors' knowledge, this study has developed the first measure to enable residents and carers to give feedback on contributory factors to safety incidents within care homes using established measure development science including Delphi, cognitive testing and consensus methods. The RMOS is therefore a theory‐ and evidence‐based measure, which utilized items from existing validated measures,^8^, ^37^ and from a recently completed systematic review.^17^ The RMOS enables residents (and/or their carers) to identify factors that could contribute to safety incidents, to enable care home settings to take meaningful action to prevent harm.

## AUTHOR CONTRIBUTIONS

Natasha Tyler, Sally Giles and Maria Panagioti conceived the design of the study, conducted the analysis and provided oversight to the study. Bethany Shears managed the Delphi rounds and recruitment. Claire Planner devised the think aloud interview schedule and conducted think aloud interviews/analysis (with Natasha Tyler and Sally Giles). Andrea Hernan provided guidance in relation to the existing measures (PC PMOS and PMOS). Maria Panagioti, Sally Giles and Natasha Tyler provided oversight of the study and drafted the manuscript. All authors reviewed and refined the manuscript.

## CONFLICT OF INTEREST STATEMENT

The authors declare no conflict of interest.

## ETHICS STATEMENT

This research received a favourable ethics opinion from Camberwell St Giles Research Ethics Committee, reference number 20/PR/0098. All participants gave informed consent to be involved in the study ahead of data collection.

## Supporting information

Supplementary information.

## Data Availability

Data are available upon reasonable request.
