# Supplementary material for: Developing the Resident Measure of Safety in Care Homes (RMOS): A Delphi and Think Aloud Study
Source: Health Expect. 2023 Feb 16;26(3):1149–58. doi: 10.1111/hex.13730 (PMC10154851; doi:10.1111/hex.13730)
Supplement: Supplementary file 1 — Supplementary information. [file HEX-26--s001.docx]

**Supplementary file 1: how items developed through Delphi rounds and scores for each round**

| It**em number** | **Item wording in R1** | **Decision for Round 1** | **Item wording in R2** | **Percentage Agree** | **Percentage Disagree** | **Median** | **Decision for Round 2** | **Researchers include** | **Family/Residents include** | **Staff include** | **Decision Consensus Meeting** | **Include/Exclude Consensus Meeting** | **Final wording for Think Aloud** |
| --- | --- | --- | --- | --- | --- | --- | --- | --- | --- | --- | --- | --- | --- |
| 1 | I always felt that staff listened to me about my concerns | Include in R2 | I have always felt that staff listen to me about my concerns' | 93% | 0% | 9 | Include | 100% | 89% | 90% | Include | Include | I always felt that staff listened to me about my concerns |
| 2 | I am given the option to involve my family or loved ones in care planning meetings | Include but reword | 'I am given the option to involve my family or loved ones in my care' | 100% | 0% | 9 | Include | 100% | 100% | 100% | Include | Include | I am given the option to involve my family or loved ones in my care |
| 3 | I was always treated with dignity and respect | Include in R2 | 'I was always treated with dignity and respect' | 96% | 4% | 9 | Include | 88% | 100% | 100% | Amend | Include | I was always treated with dignity and respect (i.e. didn't overheard staff talking about me, helped me to clean myself, asked my consent before washing me, knocked before entering room and not going through belongings uninvited, addressed me properly, chose own clothing/meals) |
| 4 | Information regarding results or medication changes occurring during my last hospital stay was available to care homes staff | Include in R2 | 'Information regarding results or medication changes occurring during my last hospital stay was available to care home staff' | 96% | 0% | 9 | Include | 100% | 89% | 100% | Amend | Include | Information regarding my care during my last hospital stay was available to care homes staff (e.g. results or medication changes) |
| 5 | I am aware of how to report a complaint if I am unhappy with the care I receive | Include in R2 | 'I am aware of how to report a complaint if I am unhappy with the care I receive' | 96% | 4% | 9 | Include | 88% | 100% | 100% | Amend | Include | I am aware of how to report a concern if I am unhappy with the care I receive |
| 6 | I am aware of how to raise any safety concerns regarding a staff member’s behaviour | Include in R2 | 'I am aware of how to raise any safety concerns regarding a staff member's behaviour' | 96% | 0% | 9 | Include | 88% | 100% | 100% | Amend | Include | I’m aware of how to raise a concern if a staff members behaviour makes me feel unsafe |
| 7 | I felt connected with family or friends whilst in the care home | Include but reword | I am able to contact my family or friends whilst in the care home' | 96% | 0% | 9 | Include | 88% | 100% | 100% | Include | Include | I am able to contact my family or friends whilst in the care home' |
| 8 | When staff talked about my care with health care professionals the information they shared was correct | Include but reword | 'Visiting health care professionals (e.g. doctors and nurses) always know important information about my care' | 93% | 0% | 9 | Include | 100% | 89% | 90% | Amend | Include | When staff talked about my care with health care professionals the information they shared was correct |
| 9 | Care home staff communicated well to all health care professionals involved in my care | Include but reword | 'Care home staff communicate clearly to all visiting health care professionals (e.g. Doctors and Nurses)' | 93% | 4% | 9 | Include | 75% | 100% | 100% | Include | Include | 'Care home staff communicate clearly to all visiting health care professionals (e.g. Doctors and Nurses)' |
| 10 | Doctors and Nurses involved in my care were experienced in my health condition | Include but reword | 'Doctors and Nurses involved in my care knew enough about my health condition' | 93% | 4% | 9 | Include | 88% | 89% | 100% | Include | Include | 'Doctors and Nurses involved in my care knew enough about my health condition' |
| 11 | After a shift change, staff knew important information about my care | Include but reword | Staff always know important information about my care' | 89% | 0% | 9 | Include | 100% | 67% | 100% | Include | Include | Staff always know important information about my care' |
| 12 | I am actively promoted to be involved in decisions on all aspects of my care | Include but reword | 'I am encouraged and supported to be involved in decisions about my care' | 89% | 4% | 9 | Include | 100% | 78% | 90% | Include | Include | 'I am encouraged and supported to be involved in decisions about my care' |
| 13 | I was able to attend hospital appointments when required | Include | 'I was able to attend hospital appointments when required' | 89% | 4% | 9 | Include | 88% | 89% | 90% | Include | Include | 'I was able to attend hospital appointments when required' |
| 14 | Staff caring for me were always able to get advice from Doctor or Nurses when required | Include but reword | 'Staff caring for me were always able to get advice from Doctors and Nurses when needed' | 89% | 4% | 9 | Include | 100% | 89% | 80% | Include | Include | 'Staff caring for me were always able to get advice from Doctors and Nurses when needed' |
| 15 | I received advice or reviews from community physiotherapists, pharmacists, dentists or other allied health care professionals when required | Include reword | 'In the day time, I was able to receive advice from Doctors and other health care professionals when needed' | 89% | 0% | 9 | Include | 100% | 89% | 80% | Amend | Include | I received advice or reviews from community physiotherapists, pharmacists, dentists or other allied health care professionals when required |
| 16 | The physical environment of the care home is comfortable for me e.g. lighting levels, noise levels, temperature and cleanliness | Include reword | 'The physical environment of the care home feels safe (e.g. good lighting levels)' | 89% | 0% | 8 | Include | 75% | 100% | 90% | Amend | Include | 'The physical environment of the care home feels safe (e.g. good lighting levels, enough space, no clutter)' |
| 17 | Care home staff always had access to information about my care needs when required | Include in R2 | 'Care home staff always have access to information about my care needs when required' | 89% | 0% | 9 | Include | 88% | 78% | 100% | Include | Include | 'Care home staff always have access to information about my care needs when required' |
| 18 | Staff always knew everything they needed to know to care for me e.g. health conditions, medications, allergies, food preferences | Include reword | 'Staff always informed about all aspects of my care (e.g. allergies and dietary requirements)' | 89% | 0% | 9 | Include | 88% | 89% | 90% | Amend | Include | I have always been given appropriate food for my dietary requirements. |
| 19 | Information about my health conditions was available to health care professionals when required e.g. discharge summaries, referral letters, test results | Include reword | 'Information about my health conditions was available to Doctors or other health care professionals when needed' | 89% | 0% | 9 | Include | 88% | 78% | 100% | Include | Include | 'Information about my health conditions was available to Doctors or other health care professionals when needed' |
| 20 | Information regarding my test results or medication changes occurring during my last hospital stay was available to my GP | Include in R2 | 'Information regarding my test results or medication changes occurring during my last hospital stay was available to my GP' | 89% | 0% | 9 | Include | 100% | 89% | 80% | Amend | Include | 'Information regarding my care during my last hospital stay was available to my GP (e.g. test results or medication changes)' |
| 21 | There was always a member of staff available with knowledge/skills to perform specific tasks | Include reword | 'There is always a member of staff available with the knowledge/skills to perform specific tasks (e.g. giving out medication)' | 89% | 0% | 9 | Include | 88% | 89% | 90% | Include | Include | 'There is always a member of staff available with the knowledge/skills to perform specific tasks (e.g. giving out medication)' |
| 22 | I felt that resident safety was a top priority for staff | Include in R2 | 'I feel that resident safety was a top priority for staff' | 89% | 0% | 8 | Include | 88% | 89% | 90% | Exclude | Exclude | Exclude |
| 23 | (Answer only if you do not have a close family member or friend) I am aware of the advocacy system in the event that I am unable to make my own decisions e.g. due to dementia, become unwell | Include reword | If I become too unwell to make my own decisions, I am aware that someone can be appointed to act in my best interest' | 89% | 4% | 8 | Include | 88% | 89% | 90% | Include | Include | If I become too unwell to make my own decisions, I am aware that someone can be appointed to act in my best interest' |
| 24 | I was always given enough information about my care | Include reword | 'I am always offered enough information about my care' | 85% | 4% | 8 | Include | 88% | 89% | 80% | Include | Include | 'I am always offered enough information about my care' |
| 25 | Care home staff communicated well to one another on all aspects of my care | Include in R2 | 'Care home staff communicate well to one another on all aspects of my care' | 85% | 4% | 9 | Include | 75% | 78% | 100% | Include | Include | 'Care home staff communicate well to one another on all aspects of my care' |
| 26 | I was able to receive out-of-hours medical advice when required | Include reword | 'At night, I am able to receive advice from a Doctor when needed' | 85% | 4% | 8 | Include | 88% | 89% | 80% | Exclude | Exclude | Exclude |
| 27 | The care home is very clean | Include | 'The care home is very clean' | 85% | 4% | 8 | Include | 75% | 89% | 90% | Include | Include | 'The care home is very clean' |
| 28 | I have been offered personal protective equipment (e.g. face masks, gloves) where appropriate | Include reword | 'I have been offered personal protective equipment (e.g. face masks, gloves) where appropriate' | 85% | 0% | 9 | Include | 75% | 89% | 90% | Exclude | Exclude | Exclude |
| 29 | Staff have been wearing appropriate personal protective equipment when interacting with me (e.g. face masks, gloves) | Include in R2 | 'Staff have been wearing appropriate personal protective equipment when interacting with me (e.g. face masks, gloves)' | 85% | 0% | 9 | Include | 75% | 89% | 90% | Amend | Include | 'Staff have been wearing appropriate PPE (personal protective equipment) when interacting with me (e.g. gloves, apron, face masks)' |
| 30 | The staff were able to resolve or prevent any mistakes or accidents that occurred | Include but reword | 'Care home staff were able to resolve any mistakes made' | 85% | 4% | 9 | Include | 88% | 78% | 90% | Include | Include | ‘Care home staff informed me of and resolved any mistakes made’ |
| 31 | I got answers to all the questions I had regarding my care | Include in R2 | 'I always receive answers to all the questions I have regarding my care' | 81% | 4% | 8 | Include | 75% | 89% | 80% | Include | Include | 'I always receive answers to all the questions I have regarding my care' |
| 32 | Staff interacted with me in a manner that I found acceptable | Include but reword | 'Staff interact with me in a professional manner' | 81% | 4% | 8 | Include | 63% | 89% | 90% | Amend | Include | Staff interacted with me in a manner that I found acceptable |
| 33 | I have been informed of any outbreaks of infections in the care home and the action plans to control further spread | Include in R2 | 'I have been informed of any outbreaks of infections in the care home and the action plans to control further spread' | 81% | 4% | 8 | Include | 75% | 89% | 80% | Include | Include | 'I have been informed of any outbreaks of infections in the care home and the action plans to control further spread' |
| 34 | I received regular reviews from my Doctor or other health care professionals regarding my health condition or medications when necessary | Include but reword | 'I receive regular reviews from my Doctor or other health care professionals' | 81% | 0% | 8 | Include | 88% | 67% | 90% | Amend | Include | 'I receive regular reviews from my Doctor or other health care professionals (e.g. nurses, podiatrists)' |
| 35 | I witnessed or experienced physical violence from staff towards residents | Include incase negatively scored | 'I witnessed or experienced physical violence from staff member towards residents' | 81% | 15% | 9 | Exclude | 100% | 78% | 70% | Exclude | Exclude |  |
| 36 | Care home staff used language that was offensive or made me upset or frightened | Include incase negatively scored | 'Care home staff used language that was offensive or made me upset or frightened' | 81% | 19% | 9 | Exclude | 88% | 78% | 80% | Include | Include | 'Care home staff used language that was offensive or made me upset or frightened' |
| 37 | I have noticed errors in my medication e.g. number of tablets, wrong tablet, missed tablet | Include in R2 as median is 9 | 'I have noticed there has been errors in my medications (e.g. number of tablets, wrong tablet, missed tablet)' | 81% | 15% | 9 | Exclude | 100% | 78% | 70% | Exclude | Exclude |  |
| 38 | Staff always seemed to know what they were meant to be doing | Include but reword | 'Staff always seemed to know what they were meant to be doing' | 78% | 4% | 8 | Excluded in R2 | 63% | 78% | 90% | Exclude | Exclude |  |
| 39 | Equipment needed for my care was always working properly | Include in R2 | 'Equipment needed for my care was always working properly' | 78% | 0% | 8 | Excluded in R3 | 75% | 78% | 80% | Include | Include | Equipment needed for my care was always working properly (hoists, call bells, walking frames) |
| 40 | I require assistance with my medications due to a large number of medications or complex care needs | Include but reword | 'I require assistance with my medications but this is not always available' | 78% | 11% | 9 | Excluded in R4 | 100% | 67% | 70% | Exclude | Exclude |  |
| 41 | Staff worked well together as a team | Include but reword | 'Staff work safely as a team' | 74% | 0% | 7 | Excluded in R5 | 50% | 89% | 80% | Exclude | Exclude |  |
| 42 | Having an ongoing relationship with health care professionals is important to me | Include in R2 | 'Having an ongoing relationship with health care professionals is important to me' | 74% | 4% | 8 | Excluded in R6 | 63% | 78% | 80% | Exclude | Exclude |  |
| 43 | I always understood the information I was given about my care | Exclude |  |  |  |  |  |  |  |  | Exclude | Exclude |  |
| 44 | A doctor changed an aspect of my care and the care home staff did not know about it | Exclude |  |  |  |  |  |  |  |  | Exclude | Exclude |  |
| 45 | Staff gave me conflicting information about my care | Exclude |  |  |  |  |  |  |  |  | Exclude | Exclude |  |
| 46 | I needed medical advice and I did not receive it in a timely manner | Exclude |  |  |  |  |  |  |  |  | Combine | Exclude |  |
| 47 | Too few staff meant that things didn’t get done on time e.g. feeding patients, administering medications | Exclude |  |  |  |  |  |  |  |  | Include and Amend | Include | There are enough staff to look after my needs |
| 48 | Care home staff were unable to get help from other care home staff when they asked for it | Exclude |  |  |  |  |  |  |  |  | Exclude | Exclude |  |
| 49 | I always know the roles of the care home staff looking after me are (e.g. nurse, care assistant etc) | Exclude |  |  |  |  |  |  |  |  | Exclude | Exclude |  |
| 50 | I didn’t know who to go to if I needed to ask a question | Exclude |  |  |  |  |  |  |  |  | Exclude | Exclude |  |
| 51 | I have always known who is responsible for my care | Exclude |  |  |  |  |  |  |  |  | Exclude | Exclude |  |
| 52 | If I was unable to attend hospital appointments then I received home visits from health care specialists (e.g. geriatricians, psychiatrists) | Exclude |  |  |  |  |  |  |  |  | Exclude | Exclude |  |
| 53 | Equipment and supplies were not always available when needed e.g. walking frames, medications | Exclude |  |  |  |  |  |  |  |  | Exclude | Exclude |  |
| 54 | I always had to wait too long for a member of staff to attend to my needs or concerns | Exclude |  |  |  |  |  |  |  |  | Include and Amend | Include | Things get done in a timely manner (e.g. to go to the toilet, medical advice) |
| 55 | Medications I have been prescribed were not always available | Exclude |  |  |  |  |  |  |  |  | Exclude | Exclude |  |
| 56 | The physical environment made it difficult for staff to do their jobs e.g. poor lighting, extreme temperatures, small office space, clutter and untidiness | Exclude |  |  |  |  |  |  |  |  | Exclude | Exclude |  |
| 57 | There was not enough space for staff to safely complete their jobs | Exclude |  |  |  |  |  |  |  |  | Exclude | Exclude |  |
| 58 | On at least one occasion, a member of staff was not able to use the necessary equipment | Exclude |  |  |  |  |  |  |  |  | Exclude | Exclude |  |
| 59 | Inexperienced care home staff seemed to find it hard when they were left to do things on their own | Exclude |  |  |  |  |  |  |  |  | Exclude | Exclude |  |
| 60 | There was equipment that staff/residents found difficult to use e.g. hoists, medical equipment | Exclude |  |  |  |  |  |  |  |  | Exclude | Exclude |  |
| 61 | I have overheard private/personal conversations about myself or other residents | Exclude |  |  |  |  |  |  |  |  | Exclude | Exclude |  |
| 62 | I witnessed physical violence from residents towards staff | Exclude |  |  |  |  |  |  |  |  | Exclude | Exclude |  |
| 63 | Other residents used language that was offensive or made me upset or frightened | Exclude |  |  |  |  |  |  |  |  | Exclude | Exclude |  |
| 64 | I needed help to clean myself or go to the toilet and I did not receive help in a timely manner | Exclude |  |  |  |  |  |  |  |  | Combine | Exclude |  |
| 65 | It was clear who was in charge of the staff | Exclude |  |  |  |  |  |  |  |  | Exclude | Exclude |  |
| 66 | Health care professionals involved in my care did not have the skills, experience or knowledge to correctly manage my health condition | Exclude |  |  |  |  |  |  |  |  | Exclude | Exclude |  |
| 67 | Staff always seemed to know what they were meant to be doing | Exclude | Duplicate of item 38 |  |  |  |  |  |  |  | Exclude | Exclude |  |
| 68 | I noticed that staff had different ways of doing the same thing e.g. performing tasks, following care plans | Exclude |  |  |  |  |  |  |  |  | Exclude | Exclude |  |
| 69 | I see a different doctor each time I have an issue with health | Exclude |  |  |  |  |  |  |  |  | Exclude | Exclude |  |
| 70 | I am never looked after by the same nurse or care home staff during the week | Exclude |  |  |  |  |  |  |  |  | Exclude | Exclude |  |
| 71 | I cannot always remember information explained to me about my care | Exclude |  |  |  |  |  |  |  |  | Exclude | Exclude |  |
| 72 | I am aware the standard of care varies between care homes | Exclude |  |  |  |  |  |  |  |  | Exclude | Exclude |  |
| 73 | I am aware that care guidelines may vary between the care homes | Exclude |  |  |  |  |  |  |  |  | Exclude | Exclude |  |

**Supplementary file 2: Changes made to items after ‘Think Aloud’ process**

| Original item | Revised item | Decision after Think Aloud |
| --- | --- | --- |
| My relative/friend is able to contact their family or friends whilst in the care home | **Residents are supported to contact their family or friends** | Amend |
| My relative/friend is given the option to involve family or loved ones in their care | **My relative/friends are involved in care** | Amend |
| **My relative/friend is always treated with dignity and respect** |  | Keep |
| **There are enough staff to look after the needs of my relative/friend** |  | Keep |
| Things get done in a timely manner (e.g. help with going to the toilet, medical advice when asked for) | **Things get done in a timely manner (e.g. help with going to the toilet)** | Amend |
| There is always a member of staff available with the knowledge/skills to perform specific tasks (e.g. giving out medication) | **There is always a member of staff available with the knowledge/skills to perform specific tasks** | Amend |
| My relative/friend is always given appropriate food for their dietary requirements | **My relative/friend is always given the right food for them** | Amend |
| **The care home is very clean** |  | Keep |
| **The physical environment of the care home feels safe (e.g. good lighting levels, enough space, no clutter)** |  | Keep |
| Equipment needed for my relative/friend's care always works properly (wheel chair, hoists, call bells, walking frames, sensor mats) | **All equipment needed for my relative/friend's care always works properly** | Amend |
| *These statements are about the staff that work in the care home (e.g. carers, nurses, nursing assistants, managers).* | ***These statements are about the staff that work in the care home (e.g. carers, nurses, nursing assistants, managers) rather than staff that visit from outside agencies*** |  |
| ***Care home staff*** |  |  |
| ..listen to my relative/friend about their concerns | **Listen to my relative/friend** | Amend |
| **...always know important information about my relative/friend's care** |  | Keep |
| ...interact with my relative/friend in a manner that they find acceptable | **...interact with my relative/friend in an acceptable manner** |  |
| **…communicate clearly to all visiting health care professionals (e.g. Doctors and Nurses)** | **Remove question** | Remove |
| … communicate well to one another on all aspects of my relative/friend's care | **Communicate well with one another** | Amend |
| **… use language that is offensive or makes my relative/friend upset or frightened** |  | Keep |
| **… wear appropriate PPE (personal protective equipment) when interacting with my relative/friend (e.g. gloves, apron, face masks)** |  | Keep |
| … informed my relative/friend of and resolved any mistakes made | **We are informed of mistakes made** | Amend |
| … always have access to information about my relative/friend's care needs when required | **… always have access to information about my relative/friend's care needs** | Amend |
| **… are always able to get advice from Doctors and Nurses when needed** |  | Keep |
| *These statements are about sharing information.* |  |  |
| My relative/friend is aware |  |  |
| ...of how to report a concern | **We are aware of how to report a concern** | Amend |
| …of how to raise a concern if a staff members behaviour makes them feel unsafe | **Of how to raise a concern about a member of staff** | Amend |
| **… is aware that someone can be appointed to act in their best interest, if they become too unwell to make their own decisions** | **Remove question** | Remove |
| My relative/friend has been informed of any outbreaks of infections in the care home and the action plans to control further spread | **We have been informed of any infection outbreaks and the action to control further spread** | Amend |
| My relative/friend is always offered enough information about their care | **We are always offered enough information about their care** | Amend |
| My relative/friend always receives answers to all the questions they have regarding their care | **We always receives answers to all the questions we have regarding care** | Amend |
| My relative/friend is encouraged and supported to be involved in decisions about their care | **We are encouraged and supported to be involved in decisions about care** | Amend |
| **When staff talked about my relative/friend's care with health care professionals the information they shared was correct** | **Remove question** | Remove |
| *These statements are about other healthcare professionals who do not work in the care home (e.g. Doctors, nurses, physiotherapists, podiatrists, occupational therapists, rehabilitation workers)* | *Healthcare professionals that visit the care home…..* |  |
| *Other Healthcare Professionals....* |  |  |
| **Regularly review my relative/friend's needs** | **Remove question** | Remove |
| … have all the necessary information about my relative/friend's health conditions | **Have all the necessary information** | Keep |
| **… know enough about my relative/friends health conditions** | **Remove question** | Remove |
| **My relative/friend receives advice or reviews from community physiotherapists, pharmacists, dentists or other allied health care professionals when required** | **Remove question** | Remove |
| *These statements are about hospital visits* |  |  |
| My relative/friend was able to attend hospital appointments when required | **My relative/friend is able to attend hospital appointments when required** | Amend |
| Information regarding my relative/friend's last hospital visit (e.g. test results or medication changes) were available to care home staff | **Care home staff had information about the most recent hospital visit (e.g. test results or medication changes)** | Amend |
| Information regarding my relative/friend's last hospital visit (e.g. test results or medication changes) was available to their doctor (GP) | **The GP had information about the most recent hospital visit (e.g. test results or medication changes)** | Amend |

**Supplementary File 3**

| **Domain** | **Definition** |
| --- | --- |
| **Access** | Access to care, medication or other services |
| **Communication** | Effectiveness of the exchange and sharing of information between staff, patients, groups, departments and services |
| **Continuity of care** | the extent to which a person experiences an ongoing relationship with a clinical team or member of a clinical team and the coordinated clinical care that progresses smoothly as the patient moves between different parts of the health service. |
| **Dignity and respect** |  |
| **Equipment (design and function)** | Availability and functioning of equipment and supplies |
| **External policy context’** | Nationally driven policies / directives that impact on the level and quality of resources available to hospitals |
| **Information flow** | The flow of information between different healthcare settings and/or different health care professionals |
| **Organisation and Care Planning** | Availability and/or knowledge of staff, treatment and/or care |
| **Patient related factors** | Patient factors refer to those features of the patient that make caring for them more difficult and therefore more prone to error. These might include abnormal physiology, language difficulties, personality characteristics (e.g. aggressive attitude). |
| **Physical environment’** | Features of the physical environment that help or hinder safe practice |
| **Primary – Secondary Care Interface** | Any issues experienced around the interface between primary and secondary care services |
| **Task performance** | Any factor related to the working of different professionals within a group which they may be able to change to improve patient safety |
| **Team factors** | Any factor related to the working of different professionals within a group which they may be able to change to improve patient safety |
| **Training and education** | Access to correct, timely and appropriate training both specific (e.g. Task related) and general (e.g. Organisation related) |
| **Can't code** | Please use this if you are unable to code the raw data |
| **Other** | Please use this for any new categories, and specify in the next column what they are |
